# Strategies for Enhancing the Permeation of CNS-Active Drugs through the Blood-Brain Barrier: A Review

**DOI:** 10.3390/molecules23061289

**Published:** 2018-05-28

**Authors:** Isra’ Zeiadeh, Anas Najjar, Rafik Karaman

**Affiliations:** Department of Bioorganic & Pharmaceutical Chemistry, Faculty of Pharmacy, Al-Quds University, Jerusalem P.O. Box 20002, Palestine; white.angel89@live.com (I.Z.); nash.najjar@gmail.com (A.N.)

**Keywords:** blood brain barrier, drug penetration strategies, monoclonal antibody, peptide-vector, nanoparticles, prodrugs

## Abstract

*Background:* The blood brain barrier (BBB) is a dynamic and functional structure which poses a vast challenge in the development of drugs acting on the central nervous system (CNS). While most substances are denied BBB crossing, selective penetration of substances mainly occurs through diffusion, carrier mediated transport, or receptor mediated transcytosis. *Methods:* Strategies in enhancing BBB penetration have been reviewed and summarized in accordance with their type of formulation. Highlights in monoclonal antibodies, peptide-vectors, nanoparticles, and simple prodrugs were included. *Conclusion:* Nanoparticles and simple prodrugs, for example, can be used for efficient BBB penetration through inhibition of efflux mechanisms, however, monoclonal antibodies are the most promising strategy in BBB penetration. Close follow-up of future development in this area should confirm our expectation.

## 1. Introduction

The blood brain barrier (BBB) is a structural and functional barrier which protects and maintains a highly controlled environment for central nervous system (CNS) neurons. The BBB lines brain and spinal cord capillaries with endothelial cells, astrocytes, pericytes, microglia, and muscle cells. The cells are connected by tight junctions and express a variety of receptors, transporters, and pores which allow for penetration of specific substances from the blood into the CNS. BBB tight junctions are formed by junctional adhesion molecules, occludin and claudin, and transmembrane proteins [1]. Transport across the BBB can be achieved by the transcellular and paracellular pathways. Lipophilic agents are usually transported via transcellular pathways, while hydrophilic molecules cross via paracellular pathways. Paracellular pathways are regulated by tight junctions. Reversibly opening paracellular pathways through antisense oligonucleotides and siRNA or tight junction targeting by toxins and proteins has previously been reported [2,3].

It is estimated that more than 98% of small molecule drugs and approximately 100% of large molecule drugs do not cross the BBB [4,5]. Consequently, the BBB plays a pivotal role in minimizing CNS toxin exposure, controlling immune–CNS communication, maintaining a low protein CNS environment, separating peripheral and CNS neuro-signals, and importantly, regulating ion homeostasis [6].

### BBB Transport Routes

In healthy individuals, substances have been found to cross the BBB through many pathways. The most common pathways are the following:

**A: Transmembrane diffusion**

The majority of compounds cross the BBB via this mechanism [7]. While it is nonsaturable, it depends significantly on drug physico-chemical properties, such as low molecular weight, and lipid solubility. While higher lipid solubility leads to better BBB penetration [8], it also leads to an unfavorable increase in oxidative metabolism and volume of distribution [8]. Therefore, the effects of increasing lipophilicity on BBB penetration must be balanced in light of membrane permeability and metabolism [9,10]. Lipid solubility is affected by several factors, such as charge, protein binding, and most importantly, molecular weight, which is rarely above 500–600 Da [11].

**B: Carrier mediated transport: Influx and efflux**

A large variety of transporters are present at the BBB; including influx transporters such as l-type amino acid transporter (LAT1), glucose transporter (GLUT1), monocarboxylate lactate transporter (MCT1), cationic amino acid transporter (CAT1), choline transporter (ChT), sodium-coupled glucose transporters (SGLTs), among others. Examples of efflux transporters include p-glycoprotein (P-gp), peptide transport system-6 (PTS-6), and breast cancer resistant protein (BCRP) [12,13,14,15]. While influx transporters enhance substance uptake by BBB, efflux transporters retard it. Transporters can also be energy dependent, such as P-gp, or energy independent, such as GLUT1. Transporter-mediated uptake is roughly 10-fold faster than trans-membrane diffusion [16] and can be utilized by either efflux inhibition or influx substrates to enhance drug delivery to the CNS.

**C: Transcytosis**

The most common type of transcytosis is receptor-mediated transcytosis. Several receptor transporters and their role in substance transport across the BBB have been identified. Insulin receptor (IR) provided the answer to how insulin can be found in abundance in the brain while there is no insulin mRNA in the brain [17]. Transferrin receptor (TfR) mediates the transport of iron from the blood across the BBB and apo-transferrin from the brain to the blood [18,19]. Many other receptors have also been located, such as low-density lipoprotein receptor (LDLR), neonatal Fc receptor (FcRn) [20], and leptin receptor (LepR) [21].

**D: Nasal delivery**

Also, intranasal delivery offers approaches to more direct and efficient drug delivery. Nasally administered drugs can reach the brain through direct or indirect pathways. Direct ones, i.e., the trigeminal nerve pathway and the olfactory mucosa pathway, offer approaches by which a hepatic first pass metabolism is potentially avoided as well as a direct diffusion into the brain. Indirect pathways involve the drug reaching the respiratory tract and circulating through the blood to reach the BBB classically. Although a small portion of the drug can eventually reach the brain (due to mucociliary clearance for example), intranasal delivery could provide answers to drug delivery to the brain [22,23,24].

**Figure 1:**
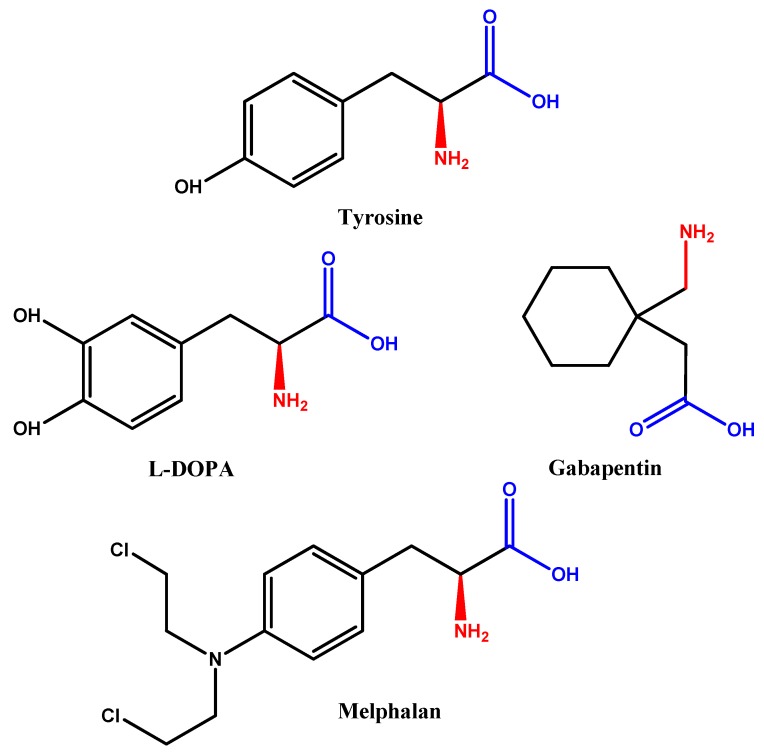
Chemical structures of tyrosine (an amino acid, LAT1 substrate), l-DOPA, Melphalan, and Gabapentin.

Drugs capable of crossing BBB have been on the market for decades and are well-known. Such drugs include l-DOPA, the metabolic precursor of dopamine, which crosses assisted by LAT1 [25]. Similarly, melphalan [26] and gabapentin [27] are substrates for LAT1 and cross through receptor-mediated endocytosis. This is due to their chemical structures mimicking that of large amino acids, like tyrosine for example, consisting of a branching structure with an amino group, a carboxyl group, and a hydrophobic side chain (Figure 1).

**Figure 2:**
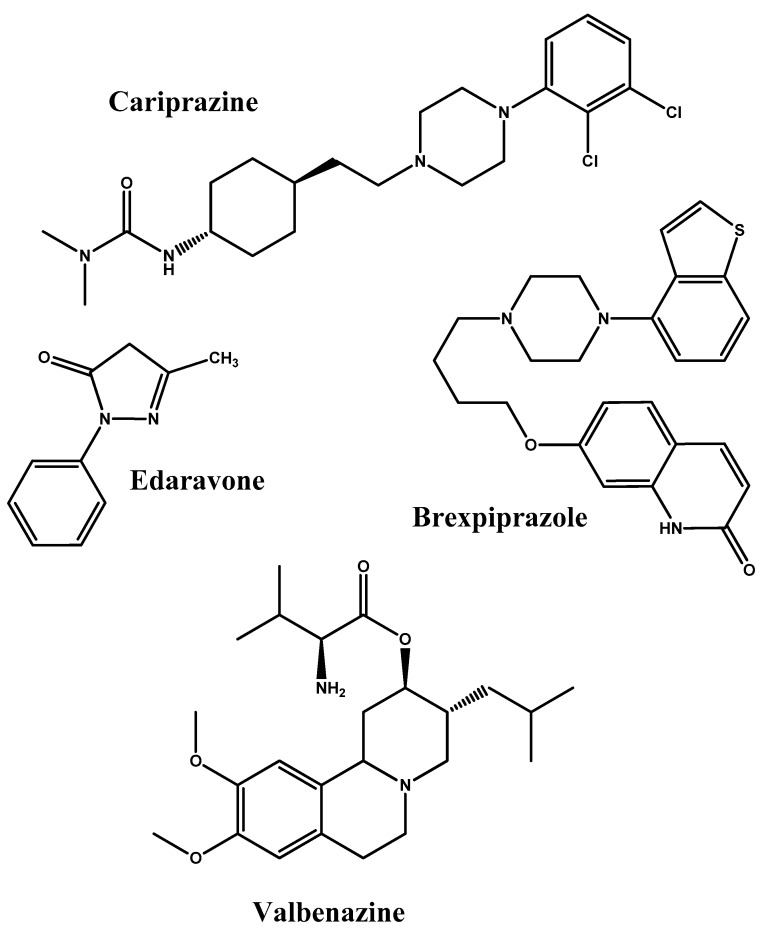
Chemical structures of cariprazine, brexpiprazole, edaravone, and valbenazine.

Moreover, new agents for brain targeted delivery are also being granted FDA approval. Recent examples include cariprazine and brexpiprazole for schizophrenia [28], daclizumab and ocrelizumab for multiple sclerosis [29], edaravone for amyotrophic lateral sclerosis [30], and valbenazine for tardive dyskinesia [31] (Figure 2). However, available treatments for neurodegenerative and CNS disorders are far from optimal. Major drawbacks from which current treatments suffer include peripheral metabolism and instability leading to the need of high doses, as well as BBB efflux and denied penetration. Many strategies are being employed to overcome the BBB penetration barrier in an attempt to potentiate treatments for those conditions. In this review, we highlight monoclonal antibodies, peptide-vectors, nanoparticles, and simple prodrugs as strategies for enhancing BBB penetration.

## 2. Drug Delivery Strategies

### 2.1. Monoclonal Antibody Strategy

While the BBB prevents penetration of the majority of arriving substances, it selectively allows others. As previously mentioned, endogenous substances such as insulin, iron, LDLs, and others are substrates for carriers located at the BBB which facilitate their penetration into the CNS. Therefore, formulation of receptor-targeted monoclonal antibodies (MAbs) is being utilized to increase penetration across the BBB.

Targeting of insulin receptors can be done using insulin, though this will lead to hypoglycaemia, therefore, monoclonal antibodies against human insulin receptors were developed. Pardridge et al. [32] developed a human insulin receptor monoclonal antibody (HIRMAb) with high affinity binding to IR (KD = 1.2 nM). Then, the extent to which this MAb could act in drug delivery was tested [33]. Biotnyl[^125^I]-Aβ^1-40^ (amyloid beta) was bound to the MAb and streptavidin. While film autoradiography determined that Aβ^1-40^ does not cross BBB, the drug-biological conjugate exhibited high brain uptake comparable to that of small molecules.

Also, HIRMAb fusion proteins were synthesized. Iduronidase (IDUA) enzyme can be used for the treatment of mucopolysaccharidosis type I (MPSI) though it does not cross the BBB. HIRMAb-IDUA enables enzyme replacement therapy by increasing BBB penetration [34,35,36]. The fusion protein AGT-181 had brain uptake of 1% ID/brain and fusion protein activity in brain predicts that an infusion dose of 1 mg/kg should restore enzyme activity to human brain. AGT-181 is currently in clinical trials for the treatment of Hurler syndrome in children; with reported treatment duration of 6 months [37].

OX26 MAb is a substrate of rat and human transferrin receptor (TfR), the reason behind its original discovery [38]. In mouse, OX26 was also found to have potential in delivering agents into mouse brain [39]. OX26 in humans was used to deliver agents across the BBB by TfR targeting such as vasoactive intestinal peptide (VIP) [40].

Bispecific antibodies (bsAb) are novel molecules with two different binding sites [24]. These molecules are still in development as BBB penetration enhancers. Recent attempts include a bsAb with TfR and BACE1 binding sites. The resulting bsAbs exhibited contrasting results due to their binding affinities to TfR. Low affinity binding to TfR resulted in increased BACE1 BBB penetration while high affinity resulted in poor penetration [24,41].

**Figure 3:**
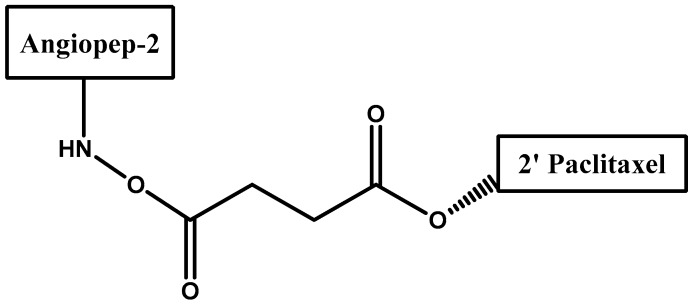
A schematic representation of ANG-1005, a conjugate between paclitaxel and Angiopep-2.

Low-density lipoprotein receptor-related protein 1 (LRP1) is another target for MAb since it is responsible for the transport of several ligands across the BBB [42,43,44]. ANG-1005, a conjugate between paclitaxel and Angiopep-2, was synthesized (Figure 3) [45]. Angiopep-2 is a derived peptide and a ligand for LRP1 [46]. Conjugated paclitaxel demonstrated significant improvement in BBB penetration vs. unconjugated paclitaxel.

Brain derived neurotrophic factor (BDNF) can be used post stroke or brain injury to provide neuroprotection and prevent neuronal death [47]. BDNF chimeric peptide was conjugated to TfRMAb through an SA-biotin linkage. The BDNF end of the peptide exerts its action on tropomyosin receptor kinase B (TRKB) receptors in the CNS for neuroprotection following brain injury while the MAb allows for targeted transport from the blood [48,49]. Hence, the resulting agent combines the monoclonal antibody strategy and the following peptide-vector strategy.

While the MAb strategy potentially has the ability to produce major BBB penetrating agents, it still, suffers from drawbacks [50]. Monoclonal antibodies targeted at specific carriers will interfere with ligand transfer, such as insulin or iron, depending on the affinity of that MAb. This drawback is expected to have worse effects as the dose of the administered agent is increased. Furthermore, immune reactions to biologic drugs have previously been reported in practice [51].

### 2.2. Peptide-Vector Strategy

Large molecules such as peptides suffer from diminished BBB penetration and efflux and thus need to be injected into the cerebrospinal fluid (CSF) if they are to be used therapeutically. Nevertheless, certain peptides can be used as P-gp inhibitors to ease the penetration of other agents.

**Figure 4:**
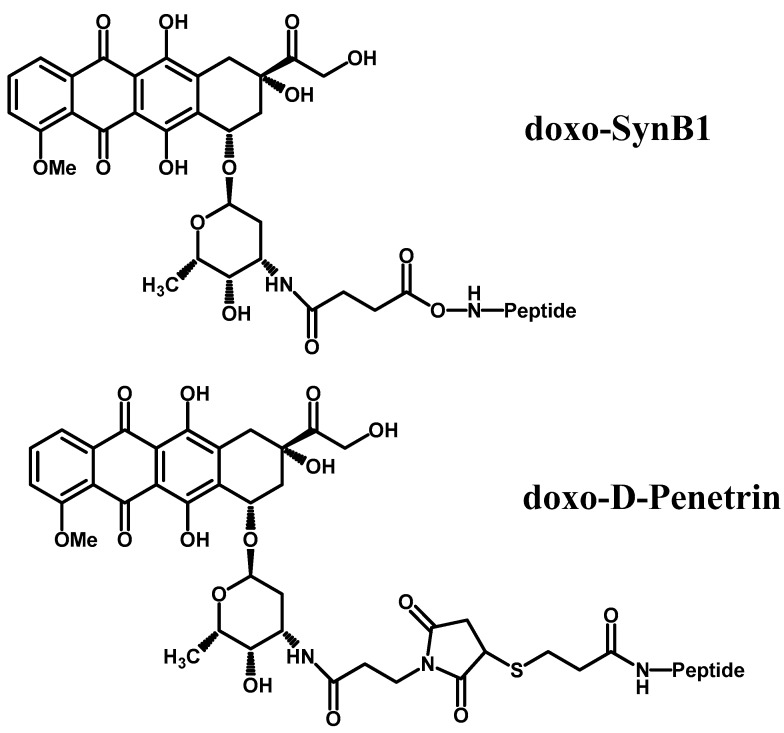
Chemical structures of doxo-SynB1 and doxo-d-Penetrin.

In a work by Rouselle et al. [52] antineoplastic doxorubicin (dox) was coupled to d-penetrin and SynB1, respectively, to enhance delivery of dox to brain (Figure 4). Dox BBB penetration was studied in rat brain perfusion is situ and by IV injection in mice. The team demonstrated that this technique resulted in a 6-fold increase in dox penetration as free dox exhibited very low uptake due to P-gp efflux while vectorized dox was able to bypass it.

Cultured neurons show that Rabies virus glycoprotein (RVG) is likely to cross the BBB through nicotinic acetylcholine receptors [53], though it does not bind to nucleic acids, and hence, is incapable of delivering siRNA. In a work by Kumar et al. [54], small interference RNA was transduced using derived RVG-9R. Specific gene silencing was achieved in mice providing protection against viral encephalitis. Results show that RVG-9R provides safe means for delivery of siRNA across the BBB.

Other peptides, such as the highly cationic peptide HIV trans-activator of transcription (TAT), were studied as possible BBB penetration enhancers. Though, while TAT’s in vitro uptake is enhanced, results still reflect negatively on its role in vivo [55,56]. Insulin-like growth factor 2 (IGF2) is also a peptide with high affinity to insulin-like growth factor receptors at the human BBB, although it suffers from high protein binding (>99%) in the blood and thus has proved impractical, as of yet, as a permeability enhancer. This was demonstrated in a fusion protein of *N*-acetyl-alpha-glucosaminidase-IGF2 (NAGLU-IGF2) [57].

### 2.3. Nanoparticles

Due to great potential that nanoparticles (NPs) possess, such as surface functionalization and decoration, size variance, drug loading capacity, immune-compatibility, and others, their applications are being investigated in several fields [58,59,60]. They can be organic, such as liposomes, or solid lipid NPs, or inorganic, such as gold or silver NPs.

B6 is a peptide sequence with affinity for TfR receptors with potential to mediate delivery across BBB [61]. Liu et al. [62] have conjugated B6 to poly(ethylene glycol)-poly(lactic acid) (PEG-PLA) NPs to allow for increased penetration and demonstrated that B6 actually plays part in cellular uptake in mouse endothelial cells.

Kim et al. [63] used chitosan NPs targeted for BBB penetration through RVG conjugation. The pluronic-based nanocarriers in combination with RVG peptide and chitosan proved efficient in increasing BBB penetration substantially while maintaining activity of the tested delivered protein. Mittal et al. [64] designed rasagiline chitosan glutamate nanoparticles. By comparing intranasal delivery, free drug solution, and IV delivery, the authors achieved significantly higher concentrations in brain following intranasal delivery.

Cheng et al. [65] tested gold NPs’ (AuNPs) ability to cross endothelial and brain tumour cells. Two types of NPs were decorated, one with TAT and another with PEG as stealth and uptake enhancers. TAT NPs exhibited markedly better penetration. AuNPs-TAT-Dox was successfully delivered in tumour-implanted mice and prevented premature death or body weight loss. Similarly, Clark and Davis [66] prepared transferrin bearing AuNPs. Transferrin was linked to the AuNPs using a linker cleavable in acidic conditions resulting in a threefold increase in BBB penetration. Silver NPs (AgNPs), on the other hand, have been found to cause concentration-dependent BBB inflammation and cytotoxicity [67], and are, therefore, unsuitable carriers for BBB penetration.

Liposomes are multi-layered spherical lipid particles which encapsulate an aqueous compartment. While the inner compartment can be used to deliver payloads, the outer lipid layers provide a shield before reaching the target site as well as sites to load lipophilic drugs. Moreover, the lipid layers can be conjugated with targeting molecules or carriers to specifically and efficiently reach the site of action, or in these cases, facilitate BBB penetration. In a study by Salvati et al. [68], an anti-transferrin receptor antibody (RI7217) was conjugated to the surface of liposomes to facilitate BBB entry. The liposomes were fluorescent and composed of sphingomyelin and cholesterol known to bind to amyloid-β. Immunoblotting revealed that the liposomes bound to TfR had higher permeability in comparison to non-decorated liposomes.

In a study by Li et al. [69], transferrin-bound carbon dots loaded with doxorubicin were synthesized. Carbon dots are a new type of biocompatible nanoparticles with unique properties [70]. Synthesized nanoparticles were composed of a carbon dot core unto which dox and transferrin were bound via carboxylic groups (CD-dox). Their efficacy was investigated in four pediatric cell lines with over-expressed TfR (CHLA-266, SJGBM2, CHLA-200, Daoy). The investigators report greater efficacy of the NPs, reducing viability by 14–45% when compared to dox alone.

The application of single walled carbon nanotubes (SWCNTs) has been studied in several areas of medicine as drug carriers, in neuro-regeneration [71], cancer, and biomedical sensors [72]. Tan et al. [73] have studied the toxicity and potential application of SWCNTs as carriers for levodopa. Results reported show that SWCNT-COOH particles exhibited sustained-release of LD over more than 20 h. Furthermore, the SWCNT-COOH-LD nanohybrid was pH activated. Importantly, cell viability was not compromised even after 72 h after treatment with the NPs. Similarly, in a recent work by Guo et al. [74], SWCNTs functionalized with lactoferrin and PEG were tested as carriers for dopamine delivery in mice. PEG coating increased stability and circulation time of the NPs while lactoferrin allowed for increased accumulation in striatum. The resulting SWCNT-PEG-Lf particles exhibit favourable properties for dopamine delivery to brain.

Other carbon nanoparticles such as Fullerenes are being explored [75,76]. Fullerenes are spherical C_60_ particles with unique geometrical shape. Though they possess unique photo-physical and antioxidant properties, their application is limited due to poor solubility in polar solvents—notably water. Therefore, conjugation of fullerenes to molecules with affinity to receptors, proteins, organelles, etc. … is crucial to their biological application. In a work by Tsao et al. [77], carboxyfullerene 20 was tested on twenty different bacterial strains. Under specific conditions, BBB permeability of *E. coli* responsible for causing meningitis was diminished. It appeared that carboxyfullerenes inhibited cytokines and other factors released by neutrophils which alter BBB permeability.

Bi et al. [78] investigated the ability of PEG-poly(lactic co-glycolic acid) (PEG-PLGA) biodegradable nanoparticles to provide efficient delivery of rotigotine to brain via nasal administration. The NPs were spherical with narrow size distribution and negative potential. Two types of NPs were prepared: non-surface modified rotigotine NPs, and rotigotine surface-modified with lactoferrin (Lf). Surface modified NPs had a size of 160 nm while unmodified rotigotine NPs were 100 nm. In vitro toxicity of the NPs was found to be low and cellular uptake was enhanced in Lf modified NPs.

Intracellular adhesion molecule 1 (ICAM-1) is a transmembrane protein expressed on cells in lysosomal disorders. Pathological factors such as oxidative stress, inflammation, and metabolic imbalance, which are characteristic of lysosomal disorders, are high up regulators of ICAM-1 expression [79]. Several works exploring targeting of ICAM-1 have been reported in the literature [80,81]. In a work by Garancho and Muro [82], polymer nanocarriers coated with ICAM-1 targeting peptide were examined. Prepared nanocarriers were endocytosed and trafficked to lysosomes, restoring levels of sphingomyelin and cholesterol within lysosomes. The authors state that the fibrinogen-derived ICAM-1 targeting peptide used in the study holds potential for lysosomal enzyme therapy.

### 2.4. Simple Prodrug Strategy

Prodrugs are molecules which upon their chemical or enzymatic activation result in an active and intended drug. This strategy has been applied widely throughout medicine in improving drug profiles and delivery [83,84,85]. Although research has taken a trend towards nanomedicine and biologicals in recent years, this strategy still can be exploited in BBB penetration due to its simplicity and vast potential.

In dopamine delivery, several prodrugs have been previously synthesized and aimed towards increased BBB penetration. Denora et al. [86] have succeeded in preparing a series of 2-phenyl-imidazopyridine-3-acetic acid substituted dopamine compounds which maintained peripheral dopamine stability while enhancing CNS delivery.

**Figure 5:**
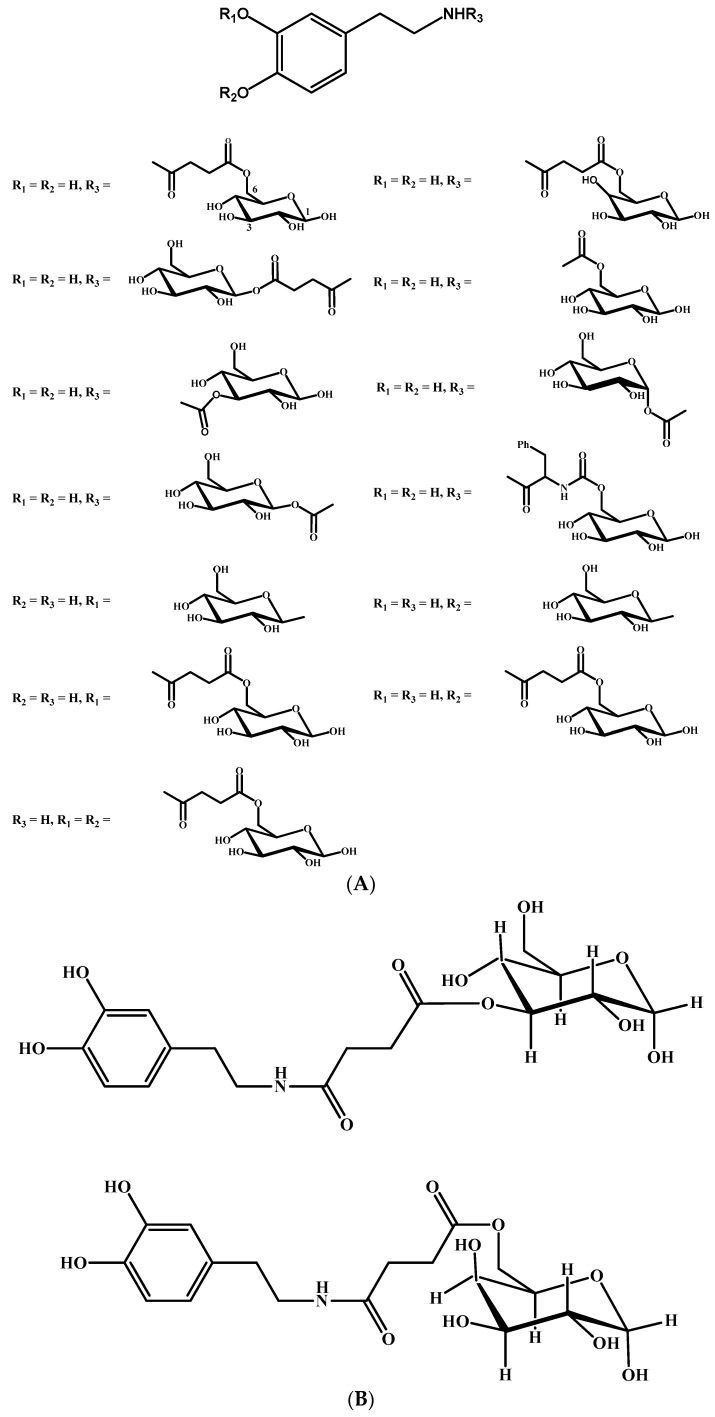
(**A**) Chemical structures of glycosylated derivatives of dopamine. (**B**) Chemical structures of glycosuccinyl derivatives of dopamine.

Due to the fact that glycosylated derivatives of drugs have the ability to increase CNS uptake by GLUT1, Fernandez et al. [87,88] have synthesized several glycosylated derivatives of dopamine using several linkers to enhance dopamine BBB penetration (Figure 5A). They report that of the derivatives tested those with conjugates substituted at C-6 were much more potent inhibitors. Ester derivatives were found too unstable in the plasma while carbamate derivatives are the prodrugs of choice. In works by Bonina et al. [89] and Ruocco et al. [90], the authors attached dopamine to glucose C-3 and C-6 also targeting GLUT1 with favourable activity when compared to l-DOPA (Figure 5B).

**Figure 6:**
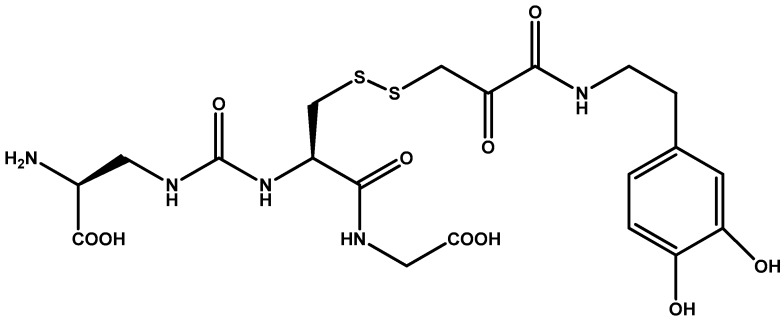
Glutathione conjugated prodrug of dopamine.

Glutathione conjugated prodrugs of dopamine were synthesized by More and Vince [91] (Figure 6) targeting BBB glutathione transporters. Their prodrugs constituted dopamine connected to glutathione as a carrier through a variety of linkages of which amide was found to have high affinity to BBB glutathione transporters.

**Figure 7:**
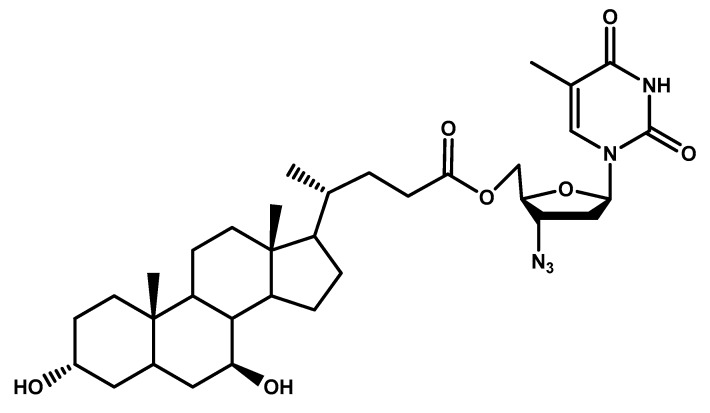
Chemical structure of AZT-UDCA prodrug.

In a work by Dalpiaz et al. [92], ursodeoxycholic acid (UDCA) was used to increase the permeability of azidothymidine (AZT) into CNS using a simple ester prodrug (Figure 7). The 5′-ester conjugate of AZT with UDCA was able to bypass CNS efflux and deliver up to twenty times more of AZT to CNS macrophages. Permeability studies were carried out on HRPE cellular monolayer.

**Figure 8:**
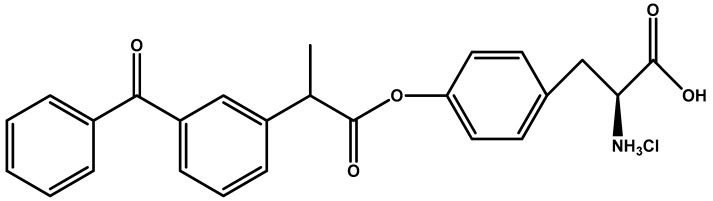
Chemical structure of ketoprofen prodrug targeted at LAT1 for CNS penetration.

Gynther et al. [93] synthesized an ester prodrug of ketoprofen and l-tyrosine (Figure 8) to deliver hydrophilic ketoprofen to CNS through LAT1. The authors tested for LAT1 binding using radiotracer [^14^C] l-leucine showing that their prodrug provided marked inhibition of tracer uptake. The ester prodrug was able to reach brain parenchyma, although the authors stated that the ester linkage present would most likely to be broken by periphery esterase.

**Figure 9:**
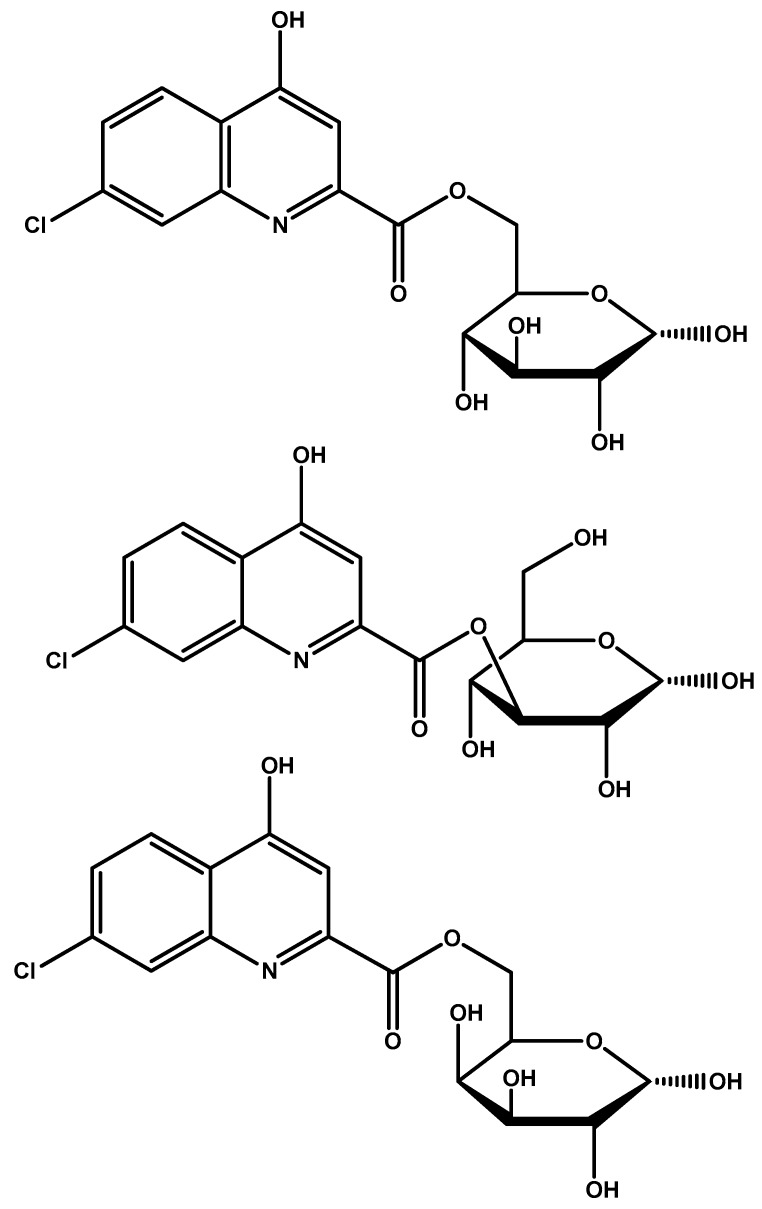
Chemical structures of glucose and galactose esters of 7-chlorokynurenic acid.

Glucose and galactose esters of 7-chlorokynurenic acid were synthesized by Battaglia et al. [94] (Figure 9) to facilitate BBB penetration through GLUT1 transporters. Following intraperitoneal injection, the delivered drug was found to have increased anti-seizure activity due to enhanced penetration. The authors state that this prodrug would be expected to undergo extensive hepatic metabolism if it were to be administered orally.

Anti-HIV drugs are good P-gp substrates and are extensively denied BBB entry. Prodrug dimers were designed with P-gp inhibition in mind to increase abacavir BBB penetration. Namanja et al. [95] linked two molecules of abacavir using sulphide and ester linkages which would be removed intracellularly. The resulting dimer was shown to have P-gp inhibitory action resulting in increased BBB penetration [96]. Linking two identical or different P-gp substrates through simple linkages is of great potential since it can allow for dual-drug delivery or selective P-gp inhibition through an inactive substrate while the desired drug is granted BBB entry.

The following Table 1 summarizes select strategies reported in this review.

## 3. Summary and Conclusions

Recent strategies can be categorized into monoclonal antibodies, peptide vectors, nanoparticles, and simple prodrugs (select works are reported in Table 1 above). Monoclonal antibodies possess significant potential, not only in BBB penetration but in other areas of drug design as well. Although, since they are tailored to match very specific sites, they tend to be quite tricky to design, thus hindering their discovery. In BBB penetration though, the recently approved daclizumab and ocrelizumab reflect the potential of this strategy, but, nevertheless, warrant close future follow-up to pass judgment on the success of this strategy.

Peptide vectors, we believe, are the least promising of the strategies mentioned in this review. While peptides are good substrates for BBB transporters, the design and synthesis of peptides alone is challenging, and those with loaded active agents and controlled release are especially much harder and time consuming to deal with. When it comes to complexity, both the peptide vectors and monoclonal antibodies are similarly complex to design, though the latter are clearly superior when it comes to BBB penetration.

Nanoparticles on the other hand, possess huge potential in BBB penetration as they do in other fields. Due to the vast types of possible nanoparticles, which can be organic or inorganic, surface decorated or not, bound to peptides or not, targeted or not, with one drug molecule or more, they appear to be the future of modern therapy though still far from complete. Nanoparticles rely on the continuous discovery and optimisation of drugs.

Simple prodrugs, such as esters, still are possible contenders in BBB penetration. Dimers are of interest since they combine P-gp inhibition and delivery in one molecule. Though those reported in the review use two identical molecules, dimers can be used in combining two complimentary agents, or perhaps three. Alas, similar to nanoparticles, the design of prodrugs relies on the existence of the original drug, also making them dependent on the continuous delivery of active drugs.

To conclude, and due to the continuous discovery of CNS active agents, it is obvious that BBB penetration is not the hardest of tasks facing drug designers nowadays. With strategies such as those highlighted in the review, and with the optimal agents, CNS drug delivery should produce a breakthrough within the upcoming decade.

## Figures and Tables

**Table 1:** Select works in improving CNS penetration reported in this review.

Strategy Tested	Agent	Notes	Future Outlook	Ref.
Human insulin receptor monoclonal antibody	None	High affinity and high transcytosis.	Further investigation and optimization.	[33,34]
Human insulin receptor monoclonal antibody	Iduronidase & IGG fusion protein	Clinical trials for Hurler syndrome in children.	Further results from trial.	[38]
Bispecific antibody	TfR and BACE1 binding sites	Application of dual action antibodies.	Research of binding site combinations.	[42]
Peptide vectorization	Doxorubicin with d-penetrin or SynB1	6-fold increase in doxorubicin permeation.	d-penetrin or SybB1 can both be used as BBB targeting entities for other drugs.	[53]
Transferrin decorated carbon dots	Doxorubicin	Greater efficacy vs free doxorubicin in 4 pediatric cell lines.	Drugs other than doxorubicin can be tested.	[70]
Single walled carbon nanotubes	Levodopa	Sustained release properties with low toxicity.	Application as drug carriers in BBB penetration.	[74]
Single walled carbon nanotubes coated with PEG and functionalized with lactoferrin	Dopamine	PEG coating increases stability of the NPs while lactoferrin produces favorable striatum accumulation	Testing in mice is promising. Further toxicity and kinetics studies before human application is tested.	[75]
Carboxyfullerene NPs	None	Interaction and inhibition with inflammatory factors maintains BBB integrity.	Fullerenes are unexplored with vast potential should solubility be overcome.	[78]
PEG-poly(lactic co-glycolic acid) NPs surface modified with lactoferrin	None	Administration intranasally for CNS delivery. Low toxicity and enhanced cellular uptake.	Lactoferrin modification of NPs for CNS delivery.	[79]
Glycosylated derivatives of l-DOPA prodrugs	l-DOPA	Carbamate derivatives are more stable than ester ones. Glycosylated derivatives at C-6 is better than C-3.	Glycosylation at C-6 provides better inhibition and uptake through GLUT-1	[88,89,90,91]
Dimer prodrug	Abacavir	Sulphide and ester linkages between two P-gp substrates increases BBB penetration.	Two identical or different P-gp substrates allows for dual-drug delivery.	[96]

## Abbreviations

BBB	Blood brain barrier
CNS	Central nervous system
LAT1	l-type amino acid transporter 1
GLUT1	Glucose transporter 1
MCT1	Monocarboxylate transporter 1
CAT1	Cationic amino acid transporter 1
ChT	Choline transporter
SGLT	Sodium-coupled glucose transporter
P-gp	P-glycoprotein
PTS-6	Peptide transport system 6
BCRP	Breast cancer resistant protein
IR	Insulin receptor
Tf	Transferrin
TfR	Transferrin receptor
LDLR	Low-density lipoprotein receptor
FcRn	Neonatal Fc receptor
LEPR	Leptin receptor
MAb	Monoclonal antibody
IDUA	Iduronidase
MPSI	Mucopolysaccharidosis type I
bsAb	Bispecific antibodies
LRP1	Low-density lipoprotein receptor related protein 1
BDNF	Brain derived neurotrophic factor
TRKB	Tropomyosin receptor kinase B
CSF	Cerebrospinal fluid
Dox	Doxorubicin
RVG	Rabies virus glycoprotein
TAT	Trans-activator of transcription
IGF2	Insulin-like growth factor 2
NAGLU	*N*-acetyl-alpha-glucosaminidase
NP	Nanoparticle
PEG	Poly (ethylene glycol)
PLA	Poly (lactic acid)
AuNP	Gold nanoparticle
AgNP	Silver nanoparticle
SWCNT	Single-walled carbon nanotube
CD	Carbon dot
PLGA	Poly (lactic co-glycolic acid)
Lf	Lactoferrin
UDCA	Ursodeoxycholic acid
AZT	Azidothymidine
ICAM-1	Intracellular adhesion molecule 1
